# Large-scale systematic review support for guideline development in diabetes precision medicine

**DOI:** 10.5195/jmla.2024.1863

**Published:** 2024-07-01

**Authors:** Maria Björklund, Krister Aronsson

**Affiliations:** 1 maria.bjorklund@med.lu.se, Librarian, Library & ICT, Faculty of Medicine, Lund University, Sweden; 2 krister.aronsson@med.lu.se, Librarian, Library & ICT, Faculty of Medicine, Lund University, Sweden

**Keywords:** Systematic review methodology, project management, search strategy development, role of information specialist, teamwork, online collaboration

## Abstract

**Background:**

Involving librarians as team members can lead to better quality in reviews. To improve their search results, an international diabetes project involved two medical librarians in a large-scale project planning of a series of systematic reviews for clinical guidelines in diabetes precision medicine.

**Case Presentation:**

The precision diabetes project was divided into teams. Four diabetes mellitus types (type 1, type 2, gestational, and monogenic) were divided into teams focusing on diagnostics, prevention, treatment, or prognostics. A search consultation plan was set up for the project to help organize the work. We performed searches in Embase and PubMed for 14 teams, building complex searches that involved non-traditional search strategies. Our search strategies generated very large amounts of records that created challenges in balancing sensitivity with precision. We also performed overlap searches for type 1 and type 2 diabetes search strategies; and assisted in setting up reviews in the Covidence tool for screening.

**Conclusions:**

This project gave us opportunities to test methods we had not used before, such as overlap comparisons between whole search strategies. It also gave us insights into the complexity of performing a search balancing sensitivity and specificity and highlights the need for a clearly defined communication plan for extensive evidence synthesis projects.

## BACKGROUND

Supporting researchers and clinicians in doing systematic reviews and clinical guidelines is a central task for many medical librarians, highlighting the need for libraries to develop organizational capacity and relevant competencies to support these projects at their institutions [1-3]. There are many roles for librarians in systematic reviews, not only including searching expertise but also methodological advice or suggestions of resources for training [2, 6-8]. Librarian collaboration and peer review of search strategies can lead to collegial learning and ensure quality in systematic reviews [4, 5]. While systematic review support requests from institutional researchers often emerge individually, we were invited to participate in an international large-scale project that sought to plan a series of systematic reviews for the development of clinical guidelines in diabetes precision medicine.

Precision medicine is an approach to optimize the diagnosis, prediction, prevention, or treatment of diabetes by integrating multidimensional data, accounting for individual differences. The major distinction from standard medical approaches is the use of complex data to characterize the individual's health status, predisposition, prognosis, and likely treatment response. Precision medicine also focuses on identifying patients who do not require treatment or less treatment. Relevant concepts are metabolic context, genomic variation, genes and transcripts, biomarkers and knowledge of lifestyle and environmental risk factors. There are research gaps on many aspects of precision medicine and how it can be translated into clinical practice guidelines [15].

### Project Setting

Lund University is a full-scale university with 47,000 students and 7,000 employees.

The Medical Faculty library supports 1,000 researchers and PhD students and 2,900 undergraduate students [9, 10]. Support to literature reviews and evidence-based medicine is offered to in different forms depending on context and target group. The library's systematic review service for researchers and PhD students is currently delivered by five librarians. The service was established in 2014 and continues to grow in number of supported reviews per year. The establishing of Cochrane Sweden [11] in Lund 2017 further enhanced the support to systematic reviews from the library, and led to collaboration with Cochrane Sweden on many areas, such as search method development, training and support for students and researchers. The team of librarians annually supports 45–65 literature reviews of various formats such as systematic reviews, Cochrane reviews, scoping reviews or clinical guidelines.

In fall 2020 we were approached by a diabetes researcher who was principal investigator (PI) for a large-scale international project. The project aimed to develop guidelines in precision medicine in diabetes, involving more than 100 researchers and clinicians around the world working in teams. The project was a part of Precision Medicine in Diabetes Initiative that was launched 2018 by American Diabetes Association and European Association for the Study of Diabetes [12]. A series of systematic reviews were planned as a basis for guideline development and a consensus report.

The PI had heard of Covidence as an online tool for systematic reviews and asked whether the library provided campus access. At this point, we had tried Covidence in small scale, but we did not have campus access. We were excited to hear about the extensive project and offered our help with Covidence, systematic searching, and methodological guidance. We were therefore happy to be engaged in the project and aimed to contribute to the methodological quality. At the outset of the project, we agreed with the PI to provide acknowledgement level contributions to each publication, choosing not to claim co-authorship as it would be time-consuming to engage deeper in so many reviews. Due to the pandemic in 2020, changes were made in our daily library routines where we switched to online support. This also made it possible for us to engage in a large project like this, as the physical library service was minimized.

Committing to participate in this project even at an acknowledgement contribution level required careful consideration from our library staff. However, following the onset of the COVID-19 pandemic 2020, we had minimized our investment in physical library services, which created additional staff availability to contribute to large projects such as this one. We also decided not to charge for our service to the project, as the PI and the project gave substantial financial contribution to the Covidence campus access, which then was made accessible for all staff and students at Lund University.

## CASE PRESENTATION

### Start-Up and Search Consultation Plan

Effective communication is essential in systematic review projects, and there are many challenges [16]. Being experienced in systematic review methodology, we were aware of some of the methodological challenges that could occur. We prioritized creating open channels of communication with project administrators and the PI to monitor progress and identify areas where additional help was needed. Rather than use a fixed support model, we instead let the needs of the PI and teams shape the process. We drafted a general project plan with method guidance tips and links to resources and also offered guidance on protocol development and Prospero registration. The documentation of our work was stored on project platforms where all teams could reach it. The majority of our communications with the PI, project administrators, and research team members took place online using Zoom, Teams, and SharePoint. The teams appreciated that we could transfer method questions and solutions across the teams which helped them forward in the review process.

**Figure 1:**
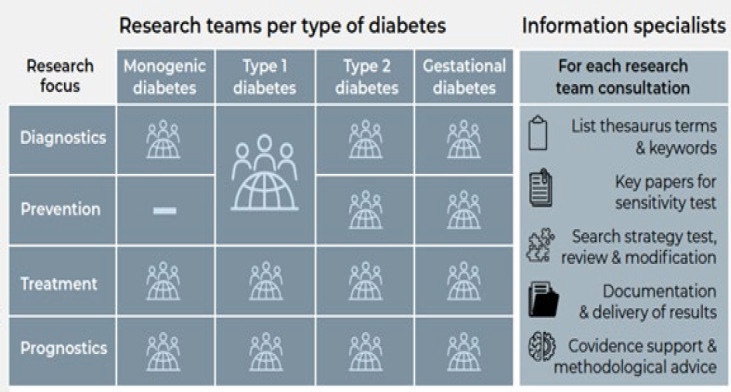
Search consultation plan and role of information specialists.

We used the team structure to plan our search consultations, communication with team coordinators and follow up, as described in Figure 1. The precision diabetes project investigated four types of diabetes: Monogenic, Type 1, Type 2, and Gestational. Each type (except for monogenic diabetes) were divided into four teams, looking at diagnostic, prevention, treatment, prognostics, creating a total of 15 possible teams. Of those teams we supported 14; the groups for Type 1 diabetes diagnostics and Type 1 diabetes prevention merged and the groups for Monogenic and gestational diabetes prognostics did not contact us for assistance.

Most groups also had subgroups, which led to multiple searches for each group. We had a startup meeting with each group to assess their needs, field questions, and to set the parameters for their searches. Some teams had developed lists of suggested terms, while other teams needed assistance with keyword generation. We then asked for key papers for each research questions for sensitivity testing. All groups gave us input on search terms, sometimes arranged within search blocks. The terms included, for example, diabetes terminology, different proteins, inhibitors, outcomes, risk assessment, diseases, pregnancy and gestation terms, substance or biomarkers. All terms were dependent on group and research area.

After conferring with the PI and the teams, and after consulting Cochrane Handbook and NICE (National Institute for Health and Care Excellence) guidelines [13, 17], it was decided that two databases, PubMed (National Library of Medicine) and Embase (Elsevier, via embase.com) were considered to be sufficient for the aim of the project. There were some teams who wanted to limit the search to just PubMed but, after consulting us, Embase was also included for better coverage.

### Building Complex Searches

The searches and search blocks were constructed and modified according to the search question. Some of the searches could have been based on models often used in systematic reviews like PICO (Patient-Intervention-Control-Outcome) or PEO (Patient/population-exposure-outcome), but for most searches these were not applicable. Instead, we worked with relevant blocks of terms corresponding to the research question. We needed to build them up in other ways, for example:

- Diabetes terms AND biomarker/marker AND outcome
- Diabetes terms AND protein AND drug
- Diabetes terms AND drug AND outcome.

The terms for diabetes were mainly the same within each section. The same Type 1 diabetes terms could be used as a search filter which were applied for most of the Type 1 diabetes searches. Controlled vocabulary was used (MeSH, Emtree), together with a Title/Abstract-search we covered most of the articles. Where the search questions were less well defined the searches would be more complex and multi-stranded searches were conducted [17]. We also used the key papers to check the sensitivity of our searches [13]. The sensitivity test included about five key papers for each search question.

We peer reviewed each other's search strategies, to make sure they were consistent across the databases but allowing for specific thesaurus terms and syntax use appropriate in each database, before running the finalized searches. Often key papers were missing, which we attributed to several different reasons, including:

- Lack of terms in one of the blocks (the most common reason)
- Paper missing from database
- No abstract available
- Older than the suggested date limitations.

Still, despite all our efforts we needed one or two unorthodox solutions to find all key papers, by adding required terms that were not related to diabetes or study design but would capture key papers.

### Overlap Searches

On request from the PI, we made overlap searches to see the overlap in between Type 1 diabetes search strategies and Type 2 diabetes search strategies, which were the diabetes types with most publications. Monogenic and gestational diabetes were more specific and gave narrower result lists. While overlap as a test method have been used to identify convergence among studies in a systematic review [18] and between databases [13], but to our knowledge overlap test of search strategies is not much explored in previous research.

**Figure 2:**
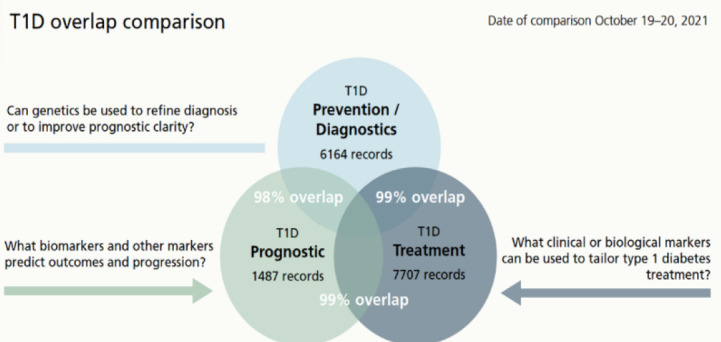
Overlap comparison search for Type 1 diabetes.

Embase was used to run the overlap test. We hypothesized that Embase would cover most of the content of our PubMed searches and provide an estimation of the overlap between the databases and within the search strategies for each diabetes type. An example of the result from an overlap search is presented in Figure 2.

The overlap searches showed that there was a substantial overlap between the searches for each diabetes type, but still searches for all teams retrieved unique papers. Our interpretation of the large overlap was that we had covered the relevant literature, with some redundancy across the searches as an expected side effect and the test revealed the extent of that overlap. The result was used by the PI and teams to collaborate more efficiently across teams. We had not tested overlap search as a method where full and complex search strategies were compared before. It was time-consuming but was highly relevant to the project.

## DISCUSSION

The large scale of the diabetes precision medicine review project gave us opportunity to test methods that we had not previously used, such as overlap comparisons of whole search strategies. Working and having meetings online were new concepts to many during the pandemic, however, large-scale projects like this would not be possible without online collaboration. The online communication platforms and tools for systematic reviews such as Covidence were essential and will continue to be core infrastructure for us. The search consultation plan (Figure 1) was essential to keep track of all the teams and their coordinators, plan our work, do status updates, and find overlaps where we could reuse previous search terms and strategies.

It was difficult to estimate how much time the activities in the review process would take for us as it depended on the teams' requirements and previous review experience. The difficulty to estimate time spent on review activities is also reported in previous research [19]. We initially spent much time on team interviews and search strategy development and testing. When core concepts of the strategies were developed, they could be reused, both within and across teams, with relevant modifications. This was time-efficient and allowed us to produce search results effectively and consistently. More time could then be dedicated to additional guidance in other parts of the review process such as full text selection and management. We did not report specific time for each project or task, but rather made a general estimation of time worked in the project.

The searches sometimes retrieved a very large number of records, especially for Type 1 diabetes and Type 2 diabetes. One challenge was to narrow down the result without losing key papers. Another problem was to find all ways of expressing important terms. Other challenges were communication and expectations. Some teams refocused their research questions and we needed to start over with searches. We had to discuss expectations of screening time, dual screening and explain general sensitivity/specificity of search results for systematic reviews, as some teams had expected narrower results, similar challenges also reported in previous studies [16, 20]. There were both advantages and disadvantages of ‘organic' management and communication compared to a fixed service model. An advantage was the flexibility to make changes without unnecessary bureaucracy. A disadvantage was some lack of transparency and expected time estimation.

We chose not to charge for our service and support. In the future, for similar large projects a support model in a twotiered fashion as suggested by McKeown [2] could be used, where methodological consultation could be billed to a funded researcher's grant [21]. As many services at a research university already operate in this explicit research core model, we anticipate that this cost-recovery model would be well-understood by institutional researchers.

Visualizing the communication plan was essential in this complex project, to ensure efficient workflow in each part, to avoid redundant work and focus on quality. We found new ways to work efficiently to maximize quality and minimize redundant work in our support to the series of systematic reviews. The complex research questions and need for complex tailored search strategies will help us recognize similar needs in the future and respond with appropriate search strategies, based on core guidelines and handbooks. Our experiences from the project helped establish the expansion of our general systematic review support to our faculty, now going beyond searching to include additional guidance in methodology and tools for systematic reviews.

The diabetes precision medicine project has so far published a consensus report and 12 of 16 planned systematic reviews. The consensus report is published in Nature Medicine [22] and the series of systematic reviews is available via Nature Communications [23].

## LIMITATIONS

There are a few noteworthy limitations related to the size of the project. Had the reviews requests been presented traditionally one by one, we might have had time and resources to use a more traditional systematic review methodology. However, then we would not have been able to deliver results within the expected time frame (2 years) of the project. In our case, the number of simultaneous reviews called for effective review management and transparency in methodological considerations. Diabetes is a research area with a significant number of publications, where our role in the project was to help the teams get a reasonable and relevant result to work with. The specific topic, precision medicine in diabetes, also led to variations in how to balance sensitivity-specificity and some variations in the use of study design filters, depending on each team's requirements and focus of research questions. We consulted handbooks, such as Cochrane Handbook [13] and NICE guidelines [17] for appropriate conduct, together with sensitivity tests of our strategies using key papers provided by the teams. Given additional time and resources, external peer review of our search strategies could have contributed to additional quality of the result.

## Data Availability

Data in the format of search strategies underlying our work are either available as supplementary material to the published papers described in this study or can be obtained from the authors. Other data associated with this article cannot be made publicly available because they contain personally identifiable information. Access to the data can be requested from the corresponding author and may be subject to Institutional Review Board restrictions.
